# 

**Published:** 1857-07

**Authors:** 


### THE SANITARY REVIEW.

### CONTENTS OF No. X.

The Sweating Sickness in England . . . . . 105
Diseased Food and Disease . . . . . . 125

**EPITOME OF SANITARY LITERATURE:**

Sewerage and Agriculture . . . . . 133
Metropolitan and Town Sewerage . . . . . 136
Animal Physics . . . . . . 137
Torquay a Resort for Consumptives . . . . *ib.*
Management of the Sick Room . . . . . 138
Statistics of Insanity . . . . . . 139
Legalised Causes of Crime . . . . . *ib.*
Climate and Rheumatism . . . . . 140
Miscellaneous Works . . . . . . *ib.*

**ORIGINAL COMMUNICATIONS:**

Hygiene of the Turkish Army. By J. N. Radcliffe, Esq. . . 141
Sanitary Legislation a First Duty of a Government. By B. Daniel, Esq. 151
Nursery Government in its Sanitary Aspects. By T. H. Barker, M.D. (Concluded from vol. ii, p. 373) . . . . . 164

**SANITARY AND SOCIAL SCIENCE.**

*Reports on Cities, Towns, and Districts.*—Huddersfield (concluded from p. 79) . . . . . . . 178
Gas Nuisances and their Removal . . . . . 191
Treatment of Persons apparently drowned . . . . 192

**PROGRESS OF EPIDEMICS in Forty Towns and Districts, contributed specially, by the Staff of Epidemic Observers** . . . 194

**SANITARY LEGISLATION.**

The Board of Health Bill . . . . . 213
Report on the Water Supply of the Metropolis . . . 214
Report on Disinfection and Deodorisation . . . . *ib.*
Sanitary Reports . . . . . . 216
Workhouses and the Medical Officers of Health . . . *ib.*

**TRANSACTIONS of the EPIDEMIOLOGICAL SOCIETY of LONDON:**

Mortality from Eruptive Fevers at Different Periods of the Year. By J. W. Tripe, M.D. . . . . . . 25
Quarterly Report of Proceedings . . . . . 39

**NOTES AND CORRESPONDENCE.**

